# Transcriptomic repertoires depict the initiation of lint and fuzz fibres in cotton (*Gossypium hirsutum* L.)

**DOI:** 10.1111/pbi.12844

**Published:** 2017-10-18

**Authors:** Haiyan Hu, Maojun Wang, Yuanhao Ding, Sitao Zhu, Guannan Zhao, Lili Tu, Xianlong Zhang

**Affiliations:** ^1^ National Key Laboratory of Crop Genetic Improvement Huazhong Agricultural University Wuhan Hubei China

**Keywords:** Cotton fibre, lint, fuzz, lncRNA, circRNAs, transcription factor

## Abstract

Cotton fibre is an important natural fibre for the textile industry. The number of fibre initials determines the lint percentage, which is an important factor for cotton fibre yield. Although fibre development has been described by transcriptomic analysis, the mechanism by which the long noncoding RNA manipulates the initiation of lint and fuzz fibres remains unknown. In this study, three lines with different lint percentages were developed by crossing Xu142 with its fibreless mutant Xu142 *fl*. We collected the epidermal cells from the ovules with attached fibres at 0 and 5 days post anthesis (DPA) from Xu142, the fibreless mutant Xu142 *fl* and the three lint percent diversified lines for deep transcriptome sequencing. A total of 2641 novel genes, 35 802 long noncoding RNAs (lncRNAs) and 2262 circular RNAs (circRNAs) were identified, of which 645 lncRNAs were preferentially expressed in the fibreless mutant Xu142 *fl* and 651 lncRNAs were preferentially expressed in the fibre‐attached lines. We demonstrated the functional roles of the three lncRNAs in fibre development via a virus‐induced gene silencing (VIGS) system. Our results showed that silencing XLOC_545639 and XLOC_039050 in Xu142 *fl* increased the number of fibre initials on the ovules, but silencing XLOC_079089 in Xu142 resulted in a short fibre phenotype. This study established the transcriptomic repertoires in cotton fibre initiation and provided evidence for the potential functions of lncRNAs in fibre development.

## Introduction

Cotton fibre, a single cell of the seed epidermis, is a major source of raw materials for the textile industry. Cotton fibre development goes through four overlapping stages: initiation, elongation, secondary cell wall synthesis and maturation (Haigler *et al*., 2012). According to mature fibre length, cotton fibres are divided into lint and fuzz. Lint fibre initiates from the day post anthesis (0 DPA) to 3 DPA, and fuzz fibre initiates from 5 DPA to 10 DPA (Lang, 1938). Many genes affecting cotton fibre initiation have been documented. Wu *et al*. used a cDNA microarray and identified 57 and 15 genes that were highly expressed in the fibre initial cells and epidermal cells of the cotton ovules, respectively (Wu *et al*., 2007). R2R3‐MYB transcription factor *GhMYB25‐like* plays an important role in the fibre initiation stage. The silencing of *GhMYB25‐like* in cotton leads to fibreless seeds similar to the fibreless mutant Xu142 *fl* (Walford *et al*., 2011). Some other R2R3‐MYB transcription factors, such as *GhMYB2*,* GhMYB25* and *GhMYB109*, also affect the development of cotton fibres (Huang *et al*., 2013; Machado *et al*., 2009; Pu *et al*., 2008). The class IV HD‐ZIP family transcription factor GaHOX1, which could rescue trichome formation of the *gl2‐2* mutant in Arabidopsis, may be involved in cotton fibre initiation (Guan *et al*., 2008). Moreover, another transcription factor from the class IV HD‐ZIP family, GhHOX3, positively controls fibre elongation in cotton (Shan *et al*., 2014). A bHLH transcription factor, GhDEL65, which could rescue trichome initiation in the *Arabidopsis gl3egl3* double mutant, was involved in regulating cotton fibre elongation (Shangguan *et al*., 2016). Moreover, two WD‐repeat genes (*GhTTG1* and *GhTTG3*) from cotton could recover trichome development in the *ttg1* mutant in Arabidopsis (Humphries *et al*., 2005). Although many transcription factors were identified in the development of cotton fibres, the entire gene network controlling cotton fibre initiation remains unclear.

The noncoding RNAs (ncRNAs), involved in a variety of developmental processes, are classified into different types based on the length of their mature products, including microRNAs (miRNAs) and small interfering RNAs (siRNAs) of 20–30 nucleotides (nt) in length; medium ncRNAs of 50–200 nt; and long noncoding RNAs (lncRNAs) with transcripts longer than 200 nt (Costa, 2005; Liu *et al*., 2013). A class of lncRNAs that link the 3′ end with the 5′ end were identified as circular RNAs (circRNAs). The functional identification of circRNAs is limited in many species. circRNAs are considered as sponge of miRNA function by binding a specific miRNA or miRNA target decoys (Hansen *et al*., 2013; Memczak *et al*., 2013). In plants, lncRNAs were identified in many species and were found to be involved in extensive biological processes. FLOWERING LOCUS C (FLC) is a key repressor of the flowering time in *Arabidopsis*, which is degraded during vernalization (Burn *et al*., 1993). Two types of lncRNAs, COOLAIR, the antisense of the orientation of *FLC*, and COLDAIR, the sense of *FLC* mRNA transcription, were found to be involved in the vernalization‐mediated FLC repression (Guttman *et al*., 2011; Liu *et al*., 2010). Phosphate is an essential mineral nutrient for plant development. *cis‐NAT*_*PHO1;2*_ is a *cis*‐natural antisense RNA that regulates *PHOSPHATE1;2* (*PHO1;2*), which is expressed as a translational enhancer in rice (Jabnoune *et al*., 2013). lncRNA IPS1 acts as a target mimic for miR399, which targets *PHOSPHATE2* (*PHO2*) to control phosphate homoeostasis (Chiou *et al*., 2006; Franco‐Zorrilla *et al*., 2007).

In cotton, a series of ncRNAs were discovered in fibre development. Wang *et al*. identified 50 566 long intergenic noncoding RNAs (lincRNAs) and 5826 long noncoding natural antisense transcripts (lncNATs) in *Gossypium barbadense* and found that a lncRNA (LINC02) was highly expressed in lint‐fuzz/lint‐fuzzless cotton compared with lintless‐fuzzless cotton (Wang *et al*., 2015). Wan *et al*. found a small interfering RNA (siRNA) produced from the *GhMML3_A12* gene that could mediate the self‐cleavage of *GhMML3_A12* transcript and produce naked seeds in the linted‐fuzzless mutant (N_1_) (Wan *et al*., 2016). In cotton, miRNA156/157 was demonstrated to function in fibre elongation, floral organ size and ovule production (Liu *et al*., 2014, 2017). miR828 and miR858 could target the MYB2 homoeologous gene in regulating cotton fibre development (Guan *et al*., 2014).

To identify functional lncRNAs in cotton lint and fuzz initiation, three representative lines with different fibre phenotypes were developed by crossing Xu142 with its fibreless mutant Xu142 *fl*. The epidermal cells of cotton ovules (attached with fibre) at 0 DPA and 5 DPA from Xu142, Xu142 *fl* and the three representative lines derived from them were collected for transcriptome sequencing. We identified 328 genes and 651 lncRNAs that were significantly up‐regulated in the epidermal cells and 131 genes and 646 lncRNAs that were significantly down‐regulated in the epidermal cells of linted‐fuzzless lines relative to the fibreless mutant in 0 DPA and 5 DPA ovules. Gene Ontology analysis showed that the differentially expressed genes and lncRNAs were enriched in the regulation of gene expression, nucleic acid metabolic process, cellular metabolic process and DNA binding. The functional validation of 14 lncRNAs during fibre initiation using a virus‐induced gene silencing (VIGS) system indicated that three lncRNAs could play important roles in regulating cotton fibre development.

## Results

### Development of cotton lines with different lint percentages through crossing Xu142 with Xu142 *fl*

**Figure 1:**
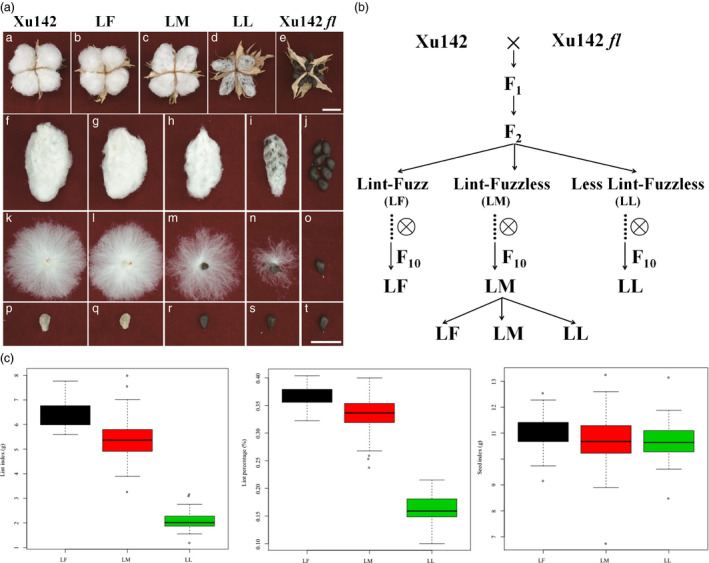
Cotton fibre morphology and lint percentage in Xu142, Xu142 *fl*, the LF (lint‐fuzz) line, the LM (lint‐fuzzless) line and the LL (less lint‐fuzzless) line. (a) The phenotype of lint and fuzz in Xu142, Xu142 *fl* and the LF, LM and LL lines (a, f, k and p: Xu142 (wild type); b, g, l and q: LF line; c, h, m and r: LM line; d, i, n and s: LL line; e, j, o and t: Xu142 *fl* (lintless‐fuzzless mutant)). Scale bar: 2 cm. (b) The genealogical tree of Xu142, Xu142 *fl* and their filial generations. LF and LL lines were pure in the F_10_ generation, but LM continued segregation into three phenotypes. (c) The seed index, lint index and lint percentage were analysed based on 251 individual plants derived from selfed seeds of an LM plant.

To dissect the molecular mechanism for fibre initiation, the fibreless cotton mutant Xu142 *fl* was crossed with its wild‐type cultivar Xu142 to produce *F*_2_ plants, which showed phenotypic variations in fibre. Three representative lines with different fibre phenotypes were collected for analysis, namely Lint‐Fuzz (LF), Lint‐Fuzzless (LM) and Less Lint‐Fuzzless (LL) (Figure 1a and b). The seeds of cotton LF lines with both lint and fuzz attached were similar to the maternal line Xu142; LM lines had lint without fuzz, and the seeds of LL lines showed less lint without fuzz (Figure 1a). These three lines were selfed up to the tenth generation, as shown in Figure 1b. Surprisingly, the LF and LL lines become pure lines with no separation for later generations, but the LM lines always produce generations containing all fibre phenotypes of LF, LM and LL, similar to the *F*_2_ population. To analyse the number of genes controlling the LM fibre phenotype, 251 seeds collected from one single plant of the tenth generation of LM lines were planted in the field, and the mature seeds attached with fibre were collected from each single plant. Phenotypic characterization showed that 56, 131 and 64 plants were categorized into the LF, LM and LL phenotypes, respectively, which was in accordance with the Mendelian genetic law with a 1:2:1 proportion (Figure S1). This suggested that the lint‐fuzzless phenotype was probably controlled by one or more linked genes. Further analysis showed that seed index had no significant difference in the three lines. The lint index and lint percentage of LL lines were obviously lower than those of the LM lines and LF lines, and higher values were found in the LF lines than in the LM lines (Figure 1c). These results indicated that the LL, LM and LF lines were excellent materials for studying the mechanism of cotton fibre development.

### Observation of fibre initiation in LL, LM, LF and their parental lines Xu142 and Xu142 *fl*

**Figure 2:**
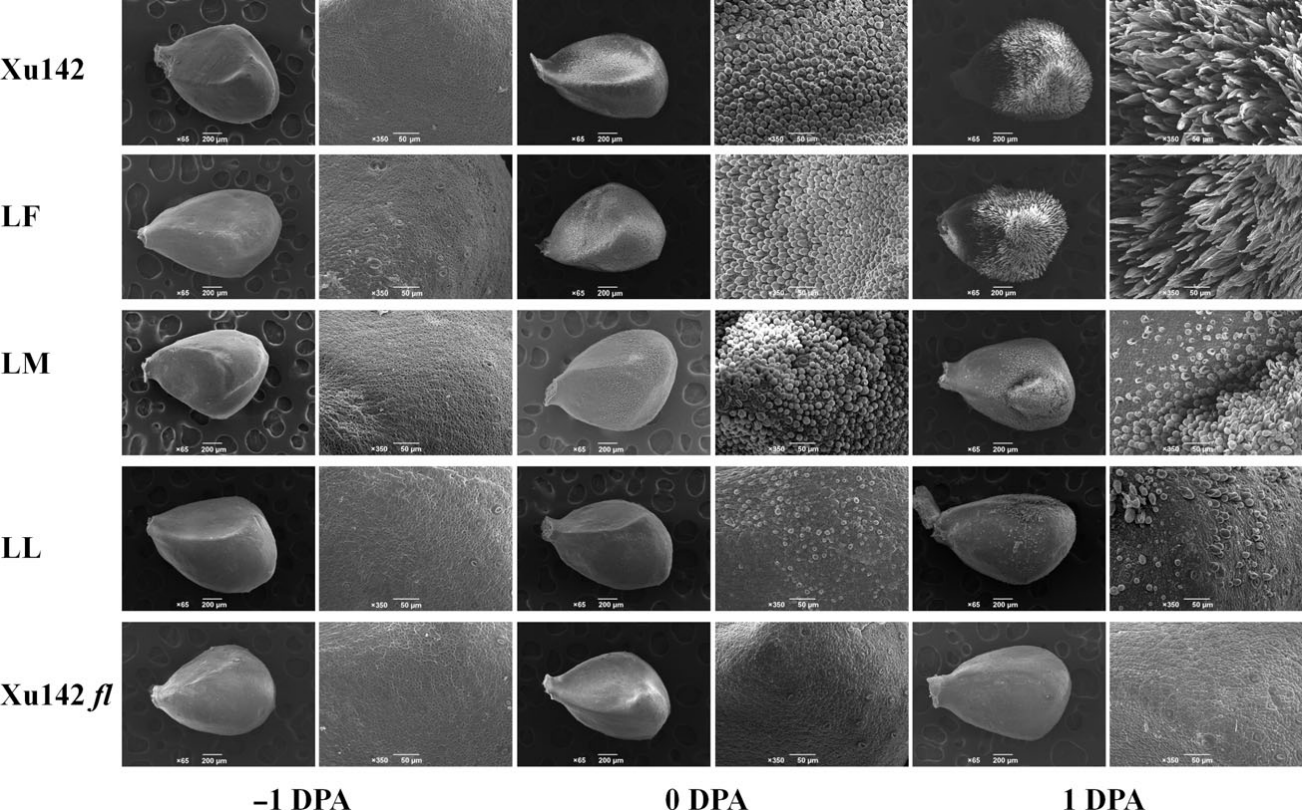
Detection of lint initiation by microscopy. The SEM photographs of −1, 0 and 1 DPA ovules of Xu142, LF, LM, LL and Xu142 *fl* (left: −1 DPA; middle: 0 DPA; right: 1 DPA). All ovules were taken from the same position in the boll; bars: 65 μm (magnified) and 200 μm.

To determine the differences in fibre initiation among the LL, LM and LF lines, −1, 0 and 1 DPA ovules from these three lines and their parental lines Xu142, Xu142 *fl* were collected for observation using scanning electron microscopy (SEM). The results showed that no fibre initials could be found in the epidermis of −1 DPA ovules from all samples. Cotton fibre initiates at 0 DPA and elongates at 1 DPA. Fibre initiation is normal on the surface around the ovules in the LF line, similar to its maternal line Xu142. However, the number of fibre initials decreased in the epidermis of the LM line, and only a few fibre initials could be found in the epidermis of the LL line. No fibre initiation was found in Xu142 *fl* (Figure 2). Moreover, fibre elongation was normal in the Xu142 and LF lines but delayed in the LM and LL lines, while no fibre elongation was observed in the Xu142 *fl* mutant (Figure 2).

### Transcriptomic repertoires of the epidermal cells in 0 DPA and 5 DPA ovules

**Figure 3:**
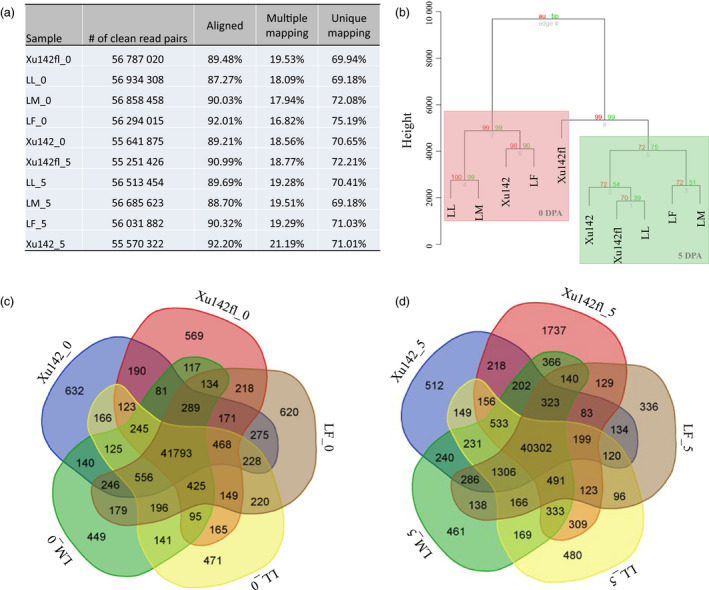
RNA sequencing data analysis. (a) Clean read pairs of the ten samples mapping to the genome. (b) Cluster analysis of ten samples was performed. (c) Venn diagram showing the number of specific genes at 0 DPA in Xu142, LF, LM, LL and Xu142 *fl*. (d) Venn diagram showing the number of specific genes at 5 DPA in Xu142, LF, LM, LL and Xu142 *fl*.

To establish the transcriptomic repertoires during cotton fibre initiation, we performed a whole transcriptome strand‐specific RNA sequencing (ssRNA‐seq) analysis using epidermal cells with attached fibre at 0 DPA and 5 DPA from the Xu142, LF, LM, LL and Xu142 *fl* lines. For each sample, we obtained at least 61 million paired‐end reads (Table S1). Raw reads were processed to obtain clean reads by removing the sequencing adaptor and filtering low‐quality reads. To remove the sequencing reads from rRNA, clean reads were mapped to the rRNA sequences in cotton using short read alignment software *SOAP aligner/SOAP2* (Li *et al*., 2009). The remaining reads were used for transcriptome assembly and expression quantification (Figure 3a). After read alignment against the genome sequence of TM‐1, we found that approximately 87.27%–92.20% of reads were aligned in these samples, of which approximately 17.94%–21.19% were multiple mapping reads and 69.18%–75.19% were unique mapping reads (Figure 3a). To uncover the transcriptomic similarity in these lines, all transcripts from each sample were used for cluster analysis based on the expression pattern and level of each transcript (Figure 3b). The results showed that the transcriptomes of 0 DPA samples from LL, LM, LF and Xu142 were clustered together. But Xu142 *fl* 0 DPA was clustered into 5 DPA group, which might due to no fibre and fuzz initials on ovules of Xu142 *fl*. On the other hand, there are no fuzz initials at 5 DPA samples of LL, LM and Xu142 *fl* either, so we speculated that ovules from Xu142 *fl* at 0 DPA were clustered together with samples from 5 DPA possibly because they all do not have fuzz initials (Figure 3b).

The number of expressed genes in each sample was analysed to identify the specifically expressed genes. We found that the majority of genes were co‐expressed in both the 0 DPA (41 793/49 876) and 5 DPA (40 302/50 468) sets of five samples. The number of genes that were expressed in two to four samples ranged from 81 to 556 in 0 DPA samples and from 83 to 1306 in 5 DPA. Notably, hundreds of genes ranging from 336 to 1737 were specifically expressed in only one 0 or 5 DPA sample (Figure 3c and d).

### Discovery of novel transcribed regions

**Figure 4:**
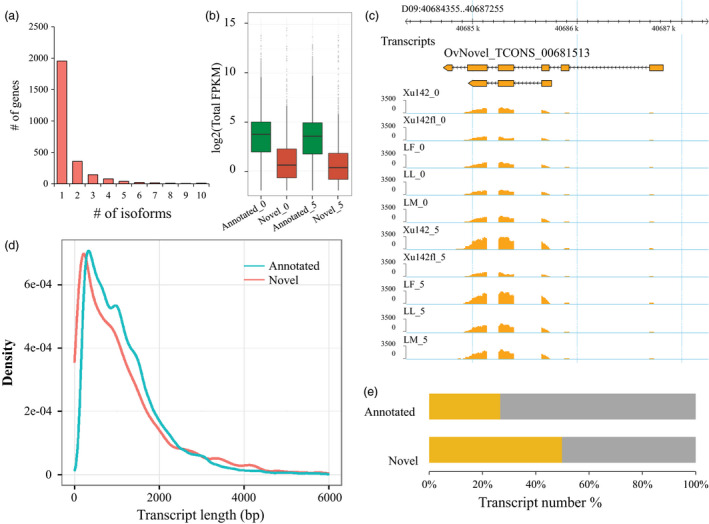
Identification and profiling of novel transcripts in cotton. (a) The transcript number of novel genes was detected. (b) The expression level of novel transcripts was lower than the annotated transcripts at 0 DPA and 5 DPA. (c) A new transcript (TCONS_00681513) mapped to the cotton genome and its lower expression level at 5 DPA for Xu142 *fl*. (d) The length of the transcripts was shorter than the annotated transcripts. (e) 26.7% of annotated transcripts were covered with the TE‐related region, and 49.8% of novel transcripts were covered with the TE‐related region.

One advantage of the full transcriptome data was to identify the novel transcribed regions in the cotton genome because these data could include all transcripts theoretically. Thus, we assembled transcripts in these samples using Cufflinks. After removing the transcripts overlapping the reference annotation, we characterized 2641 novel genes containing 4120 transcripts. We found that 74% of the genes (1954/2641) had only one transcript, 13.5% of the genes (358/2641) had two transcripts, and 12.5% of genes (329/2641) had more than two transcripts (Figure 4a).

The overall expression level of novel genes (median: 1.18 fragments per kilobase of exon per million fragments mapped (FPKM), both *P *<* *2.2 × 10^−16^, *t*‐test) was much lower than the annotated genes (median: 12.0 FPKM, both *P *<* *2.2 × 10^−16^, *t*‐test) in samples at both 0 DPA and 5 DPA (Figure 4b). An example of the novel genes (TCONS_00681513) is shown in Figure 4c. TCONS_00681513 is located in the cotton genomic region of D09:40684355–D09:40687255 and is expressed in all samples, with high expression levels in Xu142_0 and Xu142_5 (Figure 4c). This suggests that these novel transcripts could have an important function during cotton fibre development, which supplements the cotton genome information.

A comparison of transcript lengths between the annotated and novel transcripts showed that novel transcripts were shorter than annotated transcripts (Figure 4d). Interestingly, we found that 26.7% of the annotated genes and 49.8% of the novel genes were located in transposable element (TE)‐related regions, which suggested that many novel genes were associated with TEs (Figure 4e). A small number of transcripts with low expression levels were difficult to sequence and predict.

### Characterization of long noncoding RNAs and circular RNAs during cotton fibre initiation

To identify lncRNAs, the novel transcripts found by transcript assembly were subject to a potential coding test. The total novel transcripts are listed in Table S2. We found that novel coding transcripts occupied the most part (>60%), and the rest were novel noncoding transcripts (30%–40%). The proportion of novel coding or noncoding transcripts was similar in each sample for both 0 DPA and 5 DPA samples (Table S2).

**Figure 5:**
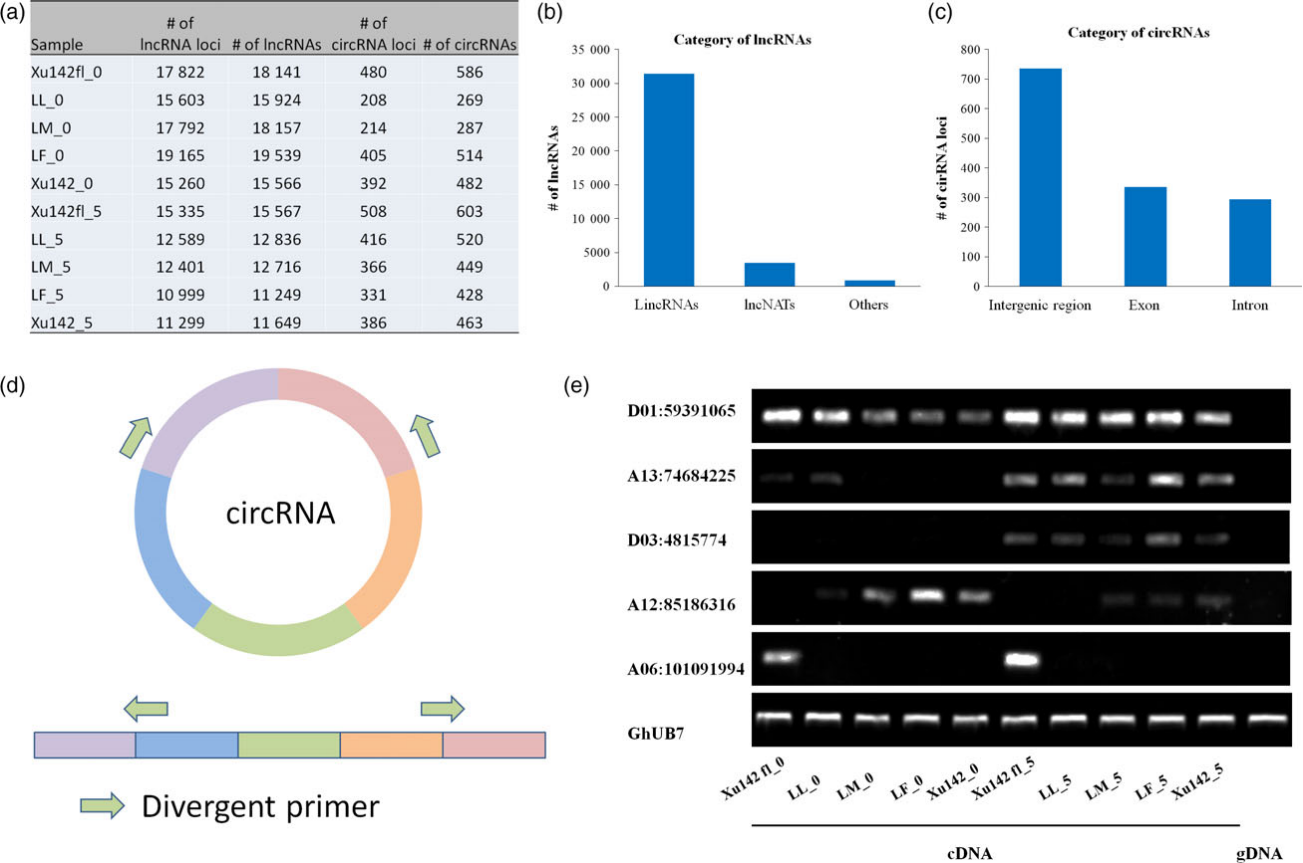
Identification and profiling of lncRNA and circRNA in cotton. (a) The number and the transcripts of lncRNA and circRNA loci were detected in ten samples. (b) The category of lncRNAs contained lincRNAs, lncNATs and others. (c) The category of circRNAs was separated in the intergenic region, exon and inter‐region. (d) Schematic diagram of circRNAs used to detect expression patterns in (e). Green arrows: divergent primers. (e) RT‐PCR analysis of circRNA expression in cDNA and gDNA.

lncRNA transcripts were identified in each sample. Most of the lncRNA loci could only transcribe one transcript, so the total number of transcripts was slightly larger than that of lncRNA loci (Figure 5a). The majority of these lncRNAs are lincRNAs (87.8%), and lncNAT only occupied 9.7% (Figure 5b).

We also identified circRNAs in our samples. A total of 2192 circRNAs were identified in ten samples of five cotton lines (Table S3). The numbers of circRNA loci and circRNA transcripts in each sample are shown in Figure 5a. More than 53.9% of circRNAs were located in the intergenic regions, 24.6% were located in the exon regions, and 21.5% were located in the intron regions (Figure 5c). Five circRNAs were randomly selected to verify ten samples using divergent primers and RT‐PCR. The result showed that circRNA D01:59391065 was expressed in all five lines and circRNAs A13:74684225 and D03:4815774 were expressed in 5 DPA epidermal cells of five lines. Moreover, circRNA A12:85186316 was detected in fibre‐specific lines (LM, LF and Xu142), and A06:101091994 was detected in fibreless lines (Xu142 *fl*), with the genomic DNA (gDNA) as the control (Figure 5d and e). circRNAs were detected in cDNA but could not be detected in gDNA, indicating the high reliability of the circRNAs. This fibre‐specific expression of circRNAs may be related to the initiation of cotton fibres, but this needs further verification.

### Transcriptomic differences between lint and fuzz fibre initiation

The specific expression of mRNAs and lncRNA was analysed in five lines. The expression levels of lncRNAs and protein‐coding genes at 0 DPA and 5 DPA for the five lines were evaluated using the FPKM values. We found that many transcription factors exhibited fibre‐specific expression patterns, such as the R2R3‐MYB transcription factors, the WD40‐repeat protein and the HD‐zip IV transcription factors. We confirmed the expression patterns of the transcription factors in the five lines using qRT‐PCR (Gh_D12G2590 (GhHOX3), Gh_A13G0689 (GhMYB106), Gh_D13G0798 (GhMYB106), Gh_D06G1439 (GhMYB‐like), Gh_D12G1628 (GhMYB25‐like), Gh_A08G0268 (GhWD40), Gh_D06G0981 (GhWD40) and Gh_D13G2002 (GhWD40)). The results showed that these transcription factors were not expressed in Xu142 *fl* and their expression levels were positively correlated with the lint percentage. Some transcription factors (GhHOX3 and GhMYB25‐like) have been reported as regulating cotton fibre development (Guan *et al*., 2008; Walford *et al*., 2011), while the rest of them may be involved in cotton fibre development.

**Figure 6:**
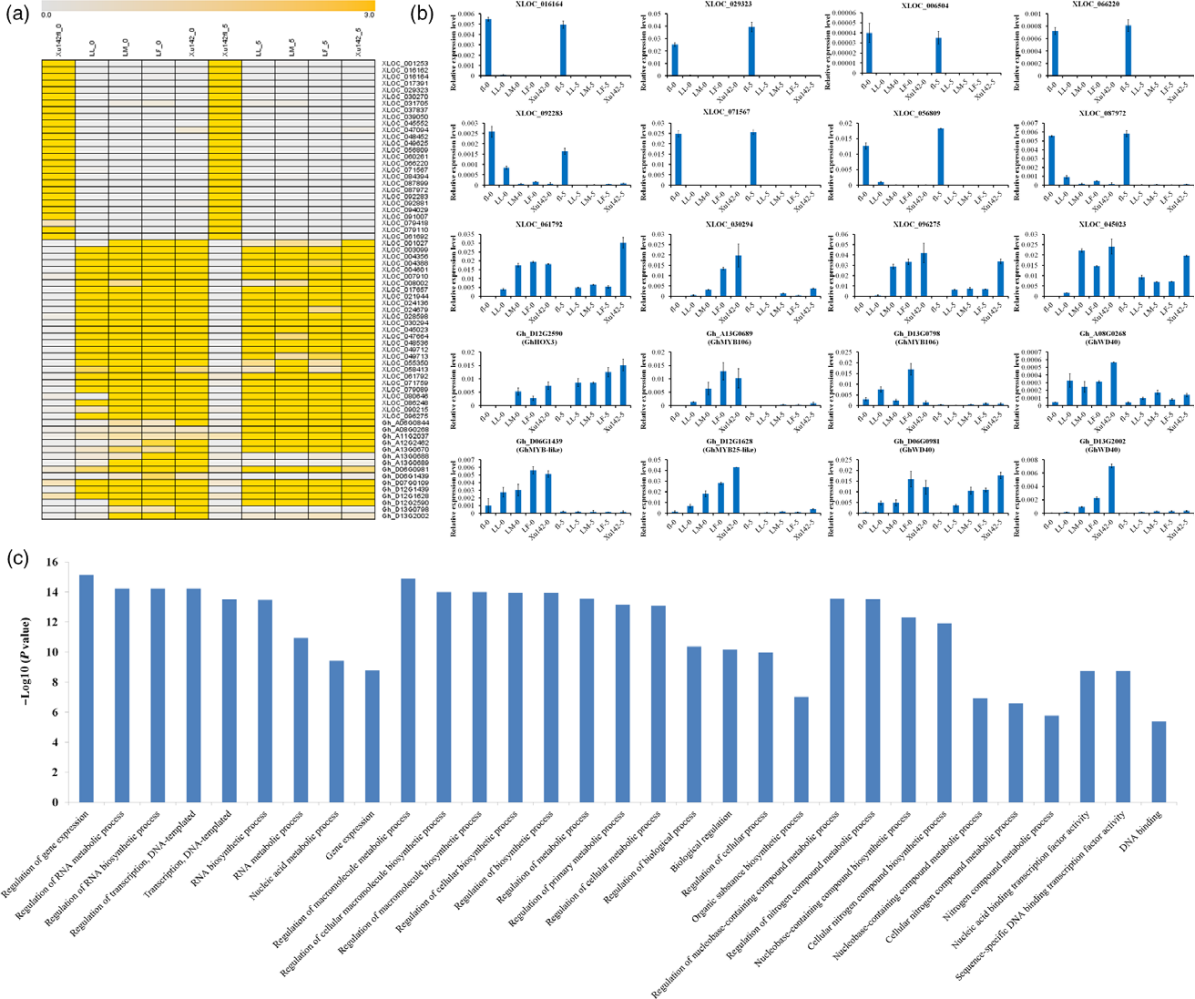
Confirmation of expression patterns of pcRNA and lncRNA using quantitative RT‐PCR (qRT‐PCR). (a) The heatmap representing the FPKM values of pcRNA and lncRNA transcript levels in 0 DPA and 5 DPA ovules. (b) The expression levels of pcRNA and lncRNA detected in ten samples using qRT‐PCR. The values shown are the means ± standard deviation of n = 3 replicates. *GhUB7* was used as the reference gene. (c) Gene ontology (GO) enrichment of fibre‐specific genes and the genes up‐ and downstream of fibre‐specific lncRNAs among Xu142, LF, LM, LL and Xu142 *fl*.

We found that some lncRNAs and genes were preferentially expressed in the fibre‐attached lines or the fibreless mutant Xu142 *fl*. The heatmap showed specific expression patterns of mRNAs and lncRNAs according to the FPKM values of ten samples (Figure 6a). Interestingly, we found that many lncRNAs are specifically expressed at 0 DPA and 5 DPA for Xu142 *fl*. To confirm the expression patterns of the lncRNAs, we randomly selected eight lncRNAs for expression validation using qRT‐PCR (XLOC_016164, XLOC_029323, XLOC_006504, XLOC_066220, XLOC_092283, XLOC_071567, XLOC_056809 and XLOC_087972), which were highly expressed in Xu142 *fl* (Figure 6b). We also detected fibre‐specific expression of lncRNAs and found that they had high expression levels in lines with high lint percentages (Figure 6b).

The interaction between the protein‐coding genes and antisense lncRNAs was predicated using *RNAplex* by complementary base pairing (Carrieri *et al*., 2012). A total of 3483 antisense lncRNA–mRNA pairs were identified in the ten samples (Table S4). Most mRNAs (76.3%) had only one paired lncRNA; however, there were also multiple pairs between mRNAs and lncRNAs. For example, two mRNAs separately interacted with eight lncRNAs, one mRNA interacted with seven lncRNAs, and nine mRNAs separately interacted with six lncRNAs (Figure S2a). In the interacted pairs, the R2R3‐MYB transcription factor Gh_D06G1439 was highly expressed in the epidermal cells of 0 DPA ovules. The expression level of Gh_D06G1439 was positively correlated with the lint percentage of five lines. TCONS_00061835 interacted with Gh_D06G1439 by complementary base pairing (Figure S2b). The expression of TCONS_00061835 was detected in ten samples with high expression in 0 DPA ovules (Figure S2c).

lncRNAs located upstream of the protein‐coding genes may overlap with the promoter regions or *cis*‐regulatory elements and could regulate the expressions of their nearby genes at the transcriptional or post‐transcriptional level (Mercer *et al*., 2009; Yazgan and Krebs, 2007). lncRNAs located downstream of protein‐coding genes may initiate transcription from the 3′ UTRs or downstream regions and may be involved in the intergenic regulatory interactions (Carninci *et al*., 2005). To predict the function of lncRNAs, we analysed the up‐ and downstream genes of specific lncRNAs. We identified the genes with fibre‐specific expression patterns, which may be involved in cotton fibre initiation. Thus, the differentially expressed genes and their up‐/downstream differentially expressed lncRNAs were used for the functional classification by Gene Ontology (GO). Web Gene Ontology Annotation Plot (WEGO) was used to present the annotations (Ye *et al*., 2006). The result showed that these genes were enriched in the regulation of gene expression, nucleic acid metabolic process, cellular metabolic process and DNA binding (Figure 6c).

### Functional analysis of fibre initiation‐related lncRNAs using virus‐induced gene silencing (VIGS)

**Figure 7:**
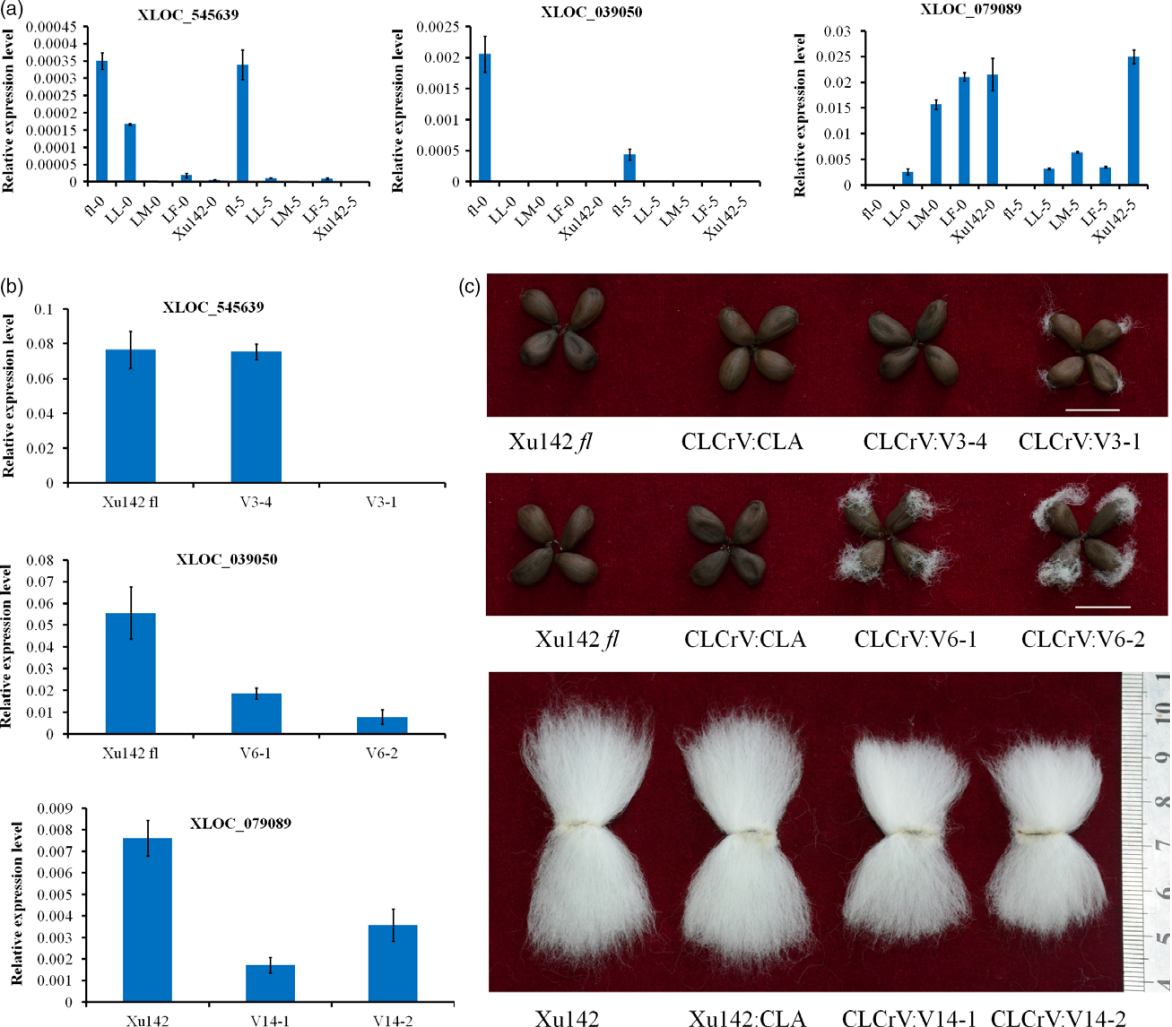
Functional characterization of three lncRNAs using the VIGS system. (a) The expression pattern of the three lncRNAs detected by qRT‐PCR. (b) Relative transcript levels of candidate lncRNAs in the silenced and control plants confirmed the effective knockdown of the targeted lncRNA. (c) Representative seeds with attached fibre from the VIGS experiment, showing the different phenotypes in the silenced lines compared with the control lines. The values shown are the means ± standard deviation of n = 3 replicates. *GhUB7* was used as the reference gene.

Many lncRNAs that may regulate cotton fibre initiation have been identified in previous studies. However, these studies failed to provide substantial functional evidence (Wang *et al*., 2015; Zou *et al*., 2016). In this study, some lncRNAs were predicted to regulate cotton fibre development according to their specific expression patterns. Expression analysis indicated that XLOC_545639 and XLOC_039050 were highly expressed in Xu142 *fl* and XLOC_079089 was highly expressed in the LL, LM, LF and Xu142 lines (Figure 7a). Therefore, we speculate that silencing of XLOC_545639 and XLOC_039050 in the fibreless mutant Xu142 *fl* would induce cotton fibre initiation and that silencing of XLOC_079089 in Xu142 would inhibit fibre initiation. To validate this possibility, a cotton leaf crumple virus (CLCrV)‐based vector, which could efficiently and persistently silence genes in cotton, was used to silence lncRNAs (Gu *et al*., 2014). An approximately 500‐bp fragment of each lncRNA was used in the VIGS system. A CLCrV:*CHLI* treatment that could block chlorophyll production was used as a positively visible control. Cotton plants, which showed no expression change in these genes, were used as a negative control. Silencing of XLOC_545639 and XLOC_039050 in the fibreless mutant Xu142 *fl* led to seeds with attached fibre in lines with expression change of genes (Figure 7b and c). The seeds of negative controls (CLCrV:*CHLI*) and fibreless mutant did not have attached fibres. Silencing of XLOC_079089 in Xu142 led to shorter fibres compared with the controls and Xu142 (Figure 7b, and c). These results showed that the three lncRNAs might be important regulators in the development of cotton fibres.

## Discussion

Cotton fibre initiation is a crucial process for determining fibre yield. Over the past decade, many genes involved in cotton fibre development have been identified. However, the mechanism of cotton fibre development is not well elucidated. In this study, a specific line was generated to explore the mechanism of cotton fibre development. The segregation ratio of the three lines produced from the LM (Lint‐Fuzzless) line was consistent with the Mendelian genetic law with a 1:2:1 proportion. The phenotypes of the three lines indicated that an incomplete dominant gene controlled fibre initiation between Xu142 and the fibreless mutant Xu142 *fl*. The lint percentages of the LM and LF (Lint‐Fuzz) lines were different, so fuzz fibre initiation may affect lint fibre initiation in the LF lines. The three different lint percentage lines were produced from one plant, so they are also good materials for mapping candidate genes.

Previous studies have identified several transcription factors involved in cotton fibre development. The R2R3‐MYB transcription factor GhMYB25‐like is an important transcription factor in regulating cotton fibre initiation. The silencing of GhMYB25‐like in cotton led to fibreless seeds, and the overexpression of GhMYB25‐like increased more fibre initials on the surface of the ovules (Hu *et al*., 2016; Walford *et al*., 2011). GhHOX3, a homoeodomain‐leucine zipper (HD‐ZIP) transcription factor, regulates cotton fibre elongation. The mature fibre length of the RNAi lines of GhHOX3 was reduced by 80% compared with the wild type (Shan *et al*., 2014). In our study, the expression of D12G1628 (GhMYB25‐like) was positively related to the number of fibre initials. The other three R2R3‐MYB transcription factors (D06G1439, A13G0689 and D13G0798) were also highly expressed in the fibre‐attached epidermal cells at 0 DPA. Three WD‐repeat genes (D06G0981, A08G0268 and D13G2002) were highly expressed in the LL, LM, LF and Xu142 lines. The expression of D12G2590 (GhHOX3) could not be detected in Xu142* fl*. The seven genes mentioned above belong to the class of WD‐repeat/bHLH/MYB transcription factors. D12G2590 is a homologous gene of GL2, an HD‐ZIP transcription factor, and acts downstream of the WD‐repeat/bHLH/MYB transcription factors. Gh_D12G2590 (GhHOX3) and Gh_D06G1439 (GhMYB‐like) have been reported to regulate cotton fibre development (Machado *et al*., 2009; Shan *et al*., 2014). The other five transcription factors ((Gh_A13G0689 (GhMYB106), Gh_D13G0798 (GhMYB106), Gh_A08G0268 (GhWD40), Gh_D06G0981 (GhWD40) and Gh_D13G2002 (GhWD40)) may be important regulators of cotton fibre development.

Previous studies have shown that several ncRNAs were involved in cotton fibre development, but their biological functions were not verified. Many miRNAs have been identified in cotton somatic embryogenesis and anthers in response to high temperature (Wei *et al*., 2013; Yang *et al*., 2013); however, only the genetic evidence for miRNA156/157 has been provided to demonstrate its important role in cotton fibre elongation (Liu *et al*., 2014). miR828 and miR858 may be involved in cotton fibre development through regulating GhMYB2A and GhMYB2D (Guan *et al*., 2014). A siRNA derived from the *GhMML3_A12* locus inhibited fibre initiation in the naked seed mutant (N_1_) plants (Wan *et al*., 2016). Zou *et al*. identified 5996 lncRNAs that contained 3510 lincRNAs and 2486 lncNATs in cotton (*Gossypium arboreum*) (Zou *et al*., 2016). Wang *et al*. obtained 50 566 lincRNAs and 5826 lncNATs transcripts in cotton and predicted that miR397 might function in regulating lignin metabolism in cotton (Wang *et al*., 2015). Lu *et al*. identified 10 820 lncRNAs from nine samples including three different environment stresses in cotton (Lu *et al*., 2016). In animals, circRNAs act as transcriptional regulators, RBP sponges and miRNA target decoys (Hansen *et al*., 2013; Memczak *et al*., 2013; Zhang *et al*., 2013). However, circRNAs have not been reported in cotton until now. In this study, we collected epidermal cells from ovules at 0 DPA and 5 DPA. Transcriptomes of the nonfibre epidermal cells and fibre epidermal cells were compared to identify the fibre‐specific or the fibreless‐specific protein‐coding genes, lncRNAs and circRNAs. Our results showed that the five lines with different lint coverage were good materials for studying cotton fibre initiation. Epidermal cells are only a small part of the ovules; the fibre‐attached epidermal cells were carefully taken from the 0 DPA and 5 DPA ovules to eliminate the influence of the embryo and endosperm cells. We systematically analysed the cotton transcripts to find protein‐coding genes, lncRNAs and circRNAs in epidermal cells of 0 DPA and 5 DPA ovules. In this study, we identified 2641 novel genes, 35 802 lncRNAs and 2262 circRNAs in the fibre‐attached epidermal cells. Some circRNAs were preferentially expressed in cotton fibre‐specific lines or fibreless‐specific lines. We experimentally validated five of the predicted circRNAs by divergent primer amplification. Of these, two were highly expressed in the epidermal cells of 5 DPA ovules, one in fibre‐specific lines and another one in fibreless‐specific lines. Therefore, we predict that circRNAs might be involved in cotton fibre development. Moreover, 3483 antisense lncRNA–mRNA pairs were identified in the ten samples. A lncRNA, TCONS_00061835, interacted with the R2R3‐MYB transcription factor Gh_D06G1439 (GhMYB‐like) by complementary base pairing. These interactions provided a new clue to analysing the function of lncRNA. According to previous studies and the predicted results, cotton fibre development might be regulated by the protein‐coding gene, lncRNA and circRNA complex network (Figure S3).

Based on our analysis, we selected seven fibre‐specific lncRNAs and seven fibreless‐specific lncRNAs for further functional analysis. According to the phenotype of the mature fibre, only two fibre‐specific lncRNAs and one fibreless‐specific lncRNA were proved to be associated with cotton fibre development using the VIGS system. Several lncRNAs were identified in cotton in previous studies (Lu *et al*., 2016; Wang *et al*., 2015; Zou *et al*., 2016), but according to the expression pattern, only lncRNA LINC02 was implicated in cotton fibre development (Wang *et al*., 2015). The first functional verification of lncRNAs was conducted in this study, which provided an insight into the lncRNAs functioning in cotton fibre development. Although we could not locate the exact gene that led to the fibreless phenotype in Xu142* fl,* we hypothesize that there is a complex network consisting of protein‐coding genes, lncRNAs and circRNAs in regulating cotton fibre development.

## Experimental procedures

### Plant materials

The cotton plant *Gossypium hirsutum* cv. Xu142 and the lintless‐fuzzless mutant Xu142 *fl* were used in this research. F_2_ plants derived from Xu142 × Xu142 *fl* cross were used to select lines with different lint coverage. According to the lint percentage phenotype, Lint‐Fuzz (LF) lines, Lint‐Fuzzless (LM) lines and Less Lint‐Fuzzless (LL) lines were selected from the F_2_ plants and planted in the experimental field for 10 years. All cotton plants used in this study were planted under standard farming conditions in Wuhan. The cotton seedlings, which were used to silence the expression of lncRNAs, were grown in a growth chamber (Conviron, Model No. ATC26) set at 25°C and a 16/8‐h light/dark cycle for 4–6 weeks. The 4‐ to 6‐week‐old VIGS cotton seedlings were transferred to a glasshouse (28–35°C by day and 20–25°C by night) under long‐day condition (16/8‐h light/dark cycle) for the entire growth period. The epidermal cells of 0 DPA ovules and epidermal cells with attached fibre of 5 DPA ovules were collected from different developmental stages using RNA*later* solution (AM7021, Thermo Fisher) by Stereo Microscope. Young leaves were collected from the VIGS cotton plants in the glasshouse. All materials were collected and immersed in liquid nitrogen and stored at −70°C until use.

### Measurement of lint percentage, lint index and seed index

To calculate the lint percentage, lint index and seed index of the LM selfed generation, 251 single plants were planted in the field. The mature bolls were separately harvested from the middle part of every plant at the same time period. One hundred seeds attached with fibre were used to record and calculate the lint percentage, lint index and seed index, and three replicates were taken for each plant.

### Observation of fibre initiation by scanning electron microscopy (SEM)

Cotton bolls at −1, 0 and 1 DPA were collected from similar positions at the same time. All ovules, which were collected from the same position of the bolls, were fixed in 2.5% (v/v) glutaraldehyde at 4°C. All ovules were dehydrated using gradient ethanol concentrations and transferred to isoamyl acetate solution. After drying, the ovules were observed and photographed using scanning electron microscopy (JSM‐6390/LV).

### Whole transcriptome library construction and sequencing

For transcriptional sequencing and gene expression level analysis, the pavement cells (epidermal cells) were collected by splitting the surface layer from the ovules under a stereomicroscope, and the RNA was extracted from the epidermal cells of ovules and leaves according to a previously reported method (Tu *et al*., 2007). After removing rRNAs from the total RNA using a kit, the transcriptome libraries were constructed using the Ribo‐Zero Kit (Illumina, USA) according to the manufacturer's instructions. Illumina HiSeq^™^ 2000 was used to sequence the mRNA and lncRNAs from the cotton ovule epidermis samples at BGI‐Tech, Shenzhen, China.

### Read mapping and transcript assembly

The assembled transcripts were compared to the reference annotation using *Cuffcompare* (Trapnell *et al*., 2012) to predict the novel transcripts. Next, we calculated the coding potential of these transcripts to identify the novel lncRNAs using Coding Potential Calculator (CPC) (Kong *et al*., 2007) and iSeeRNA (Sun *et al*., 2013).

### lncRNA and circRNA identification pipeline

Raw data were filtered to clean data by removing the adaptors and the low‐quality reads. Due to the instability of the ribosomal RNA (rRNA) removal efficiency, it is necessary to remove rRNA by alignment. Therefore, clean reads are mapped to the rRNA reference using the short read alignment software *SOAPaligner/SOAP2* (Li *et al*., 2009) to remove the remaining rRNA reads; the remaining reads were then used for transcriptome assembly and quantification. RNA‐seq data were aligned to the reference genome of *G. hirsutum* TM‐1 (AD)1 (Zhang *et al*., 2015) by *TopHat2* (Kim *et al*., 2013). The transcripts were reconstructed by *Cufflinks* (Trapnell *et al*., 2010), and several assemblies were merged by *Cuffmerge* (Trapnell *et al*., 2012). The transcripts were filtered using FPKM and a coverage threshold. The assembled transcripts were compared to the reference annotation using Cuffcompare to predict the novel transcripts. The protein‐coding transcripts and the novel lncRNA were identified from the whole transcripts using *Coding Potential Calculator* (*CPC*) (Kong *et al*., 2007) and *iSeeRNA* (Sun *et al*., 2013). *RNAplex* (Carrieri *et al*., 2012) was used to search all antisense lncRNA–mRNA duplexes with the complementary base pairing. circRNAs were identified using CIRI (Gao *et al*., 2015).

### Quantitative real‐time PCR analysis

Total RNAs were reverse‐transcribed using the SuperScript III reverse transcriptase (Invitrogen, Cat. No. 18080‐093, USA). The ABI Prism 7500 system was used to perform qRT‐PCR, with GhUBQ7 (DQ116441) as the internal control. The real‐time PCR primers are listed in Table S5.

### VIGS of three lncRNAs in cotton

A cotton leaf crumple virus (CLCrV)‐based vector was used in this research. The lengths of the silencing fragments of XLOC_545639, XLOC_039050 and XLOC_079089 were 498, 499 and 502 bp, respectively. The infusion enzyme (LN229051, Vazyme) was used to construct the silencing vectors (CLCrV:V3 CLCrV:V6 CLCrV:V14). All vector constructs were transformed into *Agrobacterium tumefaciens* strain GV3101 by electroporation. The strain GV3101 containing the different constructs was infiltrated into the cotyledons of 2‐week‐old cotton seedlings according to standard methods described by Gu *et al*. (Gu *et al*., 2014). All primers used in the VIGS experiments are listed in Table S5.

### Data access

The raw transcriptome sequencing data are available in the NCBI Sequence Read Archive (SRA) under the BioProject accession ID PRJNA266265.

## Supporting information


**Figure S1** Lint percentage of three representative lines.
**Figure S2** Protein‐coding genes interacted with lncRNAs, and the interaction model of pcRNAs and lncRNAs.
**Figure S3** The model of miRNAs, lncRNAs, circRNAs and protein‐coding genes in regulating fibre initiation.

**Table S1** Total reads of each sample.

**Table S2** The total novel transcripts of each sample.

**Table S3** All predicted circRNAs expression level.

**Table S4** Antisense lncRNA‐mRNA pairs were identified.

**Table S5** Primers used in the research.
