# Supplementary material for: Transcriptomic repertoires depict the initiation of lint and fuzz fibres in cotton (Gossypium hirsutum L.)
Source: Plant Biotechnol J. 2017 Oct 18;16(5):1002–12. doi: 10.1111/pbi.12844 (PMC5902776; doi:10.1111/pbi.12844)
Supplement: Supplementary file 1 — Figure S1 Lint percentage of three representative lines. Figure S2 Protein‐coding genes interacted with lncRNAs, and the interaction model of pcRNAs and lncRNAs. Figure S3 The model of miRNAs, lncRNAs, circRNAs and protein‐coding genes in regulating fibre initiation. [file PBI-16-1002-s005.pdf]

Supplementary Figures

(a)

	Lint-Fuzz (LF)	Lint-Fuzzless (LM)	Less Lint-Fuzzless (LL)
Lint Percentage (x%)	$34.74 \leq x \leq 40.38$	$25.40 \leq x \leq 39.05$	$10.00 < x < 21.52$
Number of single plants	56	131	64
Ratio	LF : LM : LL = 1 ： 2 ： 1		

(b)

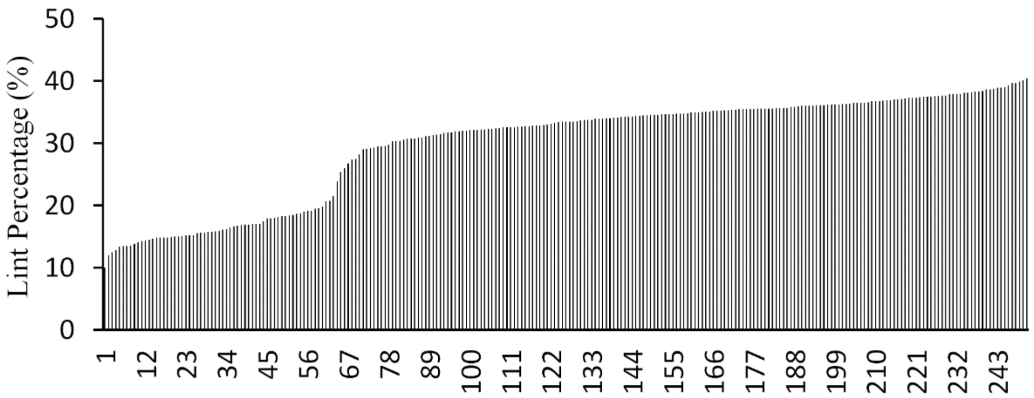

Figure S1 Lint percentage of three representative lines. (a) The lint percentage ranges and the corresponding plant numbers of three representative lines. The ratio between each line is 1:2:1. (b) Lint percentage of 251 single plants.

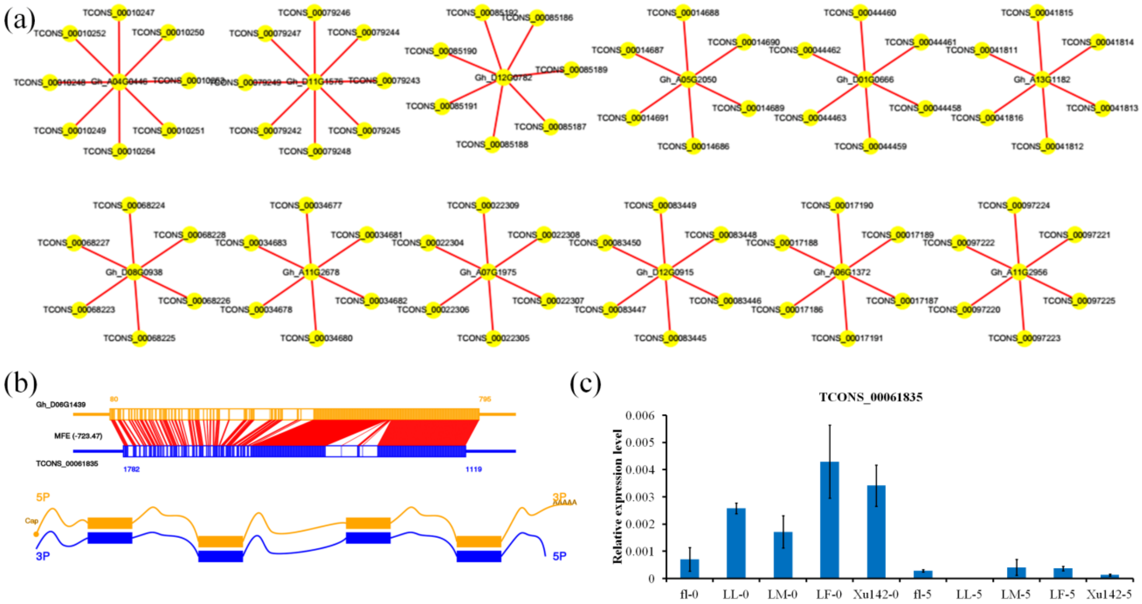

Figure S2 Protein-coding genes interacted with lncRNAs, and the interaction model of pcRNAs and lncRNAs. (a) Multiple lncRNAs interacted with one protein-coding gene. (b) Gh_D06G1439 interacted with TCONS_00061835 at the nucleic acid level. (c) The expression of TCONS_00681835 was detected in ten samples.

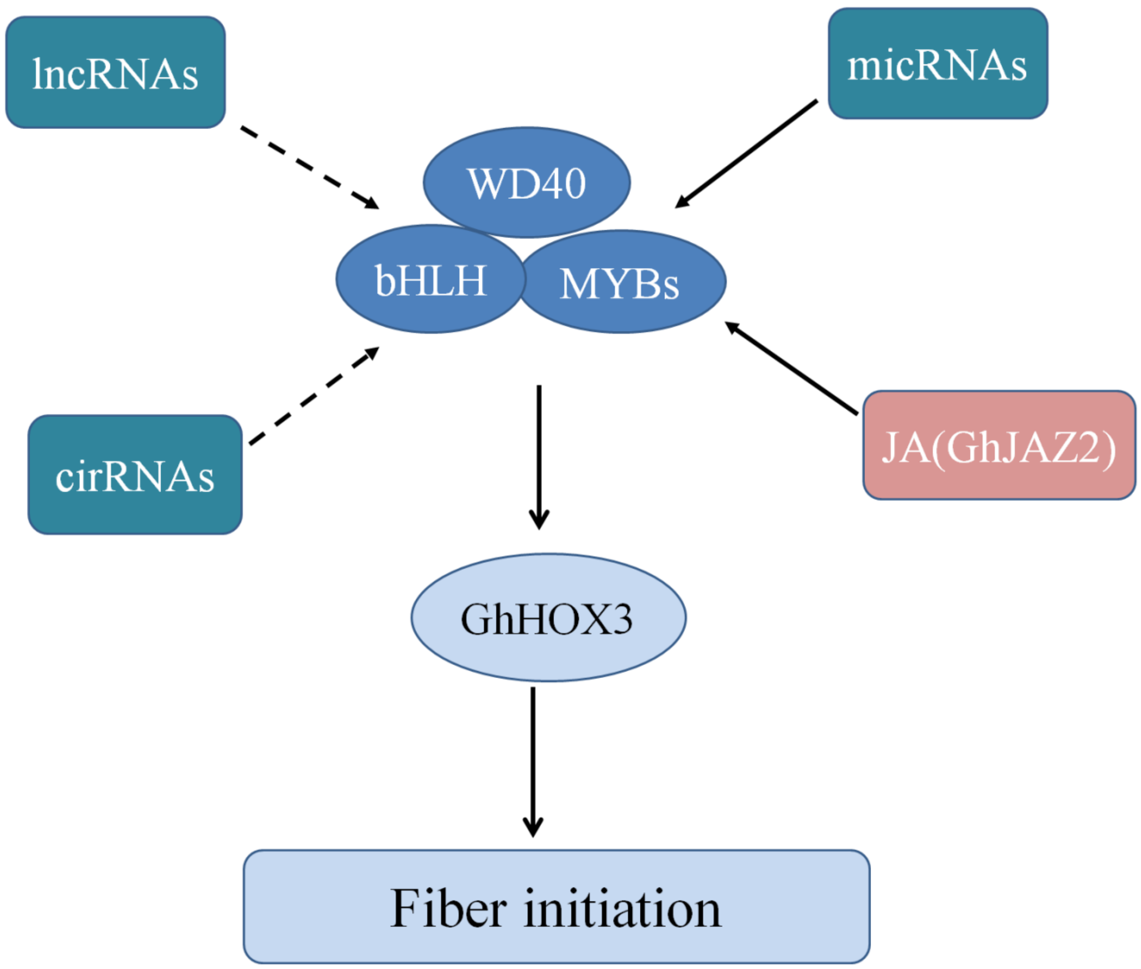

Figure S3 The model of miRNAs, lncRNAs, circRNAs and protein coding genes in regulating fiber initiation. In this network, miRNAs and GhJAZ2 affected R2R3-MYB transcription factors to regulate cotton fiber development. lncRNAs and circRNAs may target WD-repeat/bHLH/MYB complex expression to control cotton fiber development.
