# Supplementary material for: Transcriptomic repertoires depict the initiation of lint and fuzz fibres in cotton (Gossypium hirsutum L.)
Source: Plant Biotechnol J. 2017 Oct 18;16(5):1002–12. doi: 10.1111/pbi.12844 (PMC5902776; doi:10.1111/pbi.12844)
Supplement: Supplementary file 2 — Table S1 Total reads of each sample. [file PBI-16-1002-s004.docx]

Table S1 Total reads of each sample.

|  | **left reads mapping** | | | | **right reads mapping** | | | |
| --- | --- | --- | --- | --- | --- | --- | --- | --- |
| **sample** | **total reads** | **unmapped reads** | **unique mapped reads** | **multiple mapped reads** | **total reads** | **unmapped reads** | **unique mapped reads** | **multiple mapped reads** |
| LF_0 | 61926013 (100.00%) | 12654852 (20.44%) | 21916487 (35.39%) | 27354674 (44.17%) | 61934330 (100.00%) | 11632896 (18.78%) | 20218047 (32.64%) | 30083387 (48.57%) |
| LF_5 | 61651603 (100.00%) | 14742604 (23.91%) | 18589591 (30.15%) | 28319408 (45.93%) | 61659165 (100.00%) | 13899895 (22.54%) | 16915589 (27.43%) | 30843681 (50.02%) |
| LL_0 | 61961144 (100.00%) | 15172834 (24.49%) | 19212396 (31.01%) | 27575914 (44.51%) | 61970896 (100.00%) | 14259894 (23.01%) | 17488554 (28.22%) | 30222448 (48.77%) |
| LL_5 | 61746731 (100.00%) | 13838712 (22.41%) | 19647389 (31.82%) | 28260630 (45.77%) | 61760931 (100.00%) | 12964409 (20.99%) | 17952197 (29.07%) | 30844325 (49.94%) |
| LM_0 | 61904399 (100.00%) | 13506972 (21.82%) | 20771526 (33.55%) | 27625901 (44.63%) | 61917159 (100.00%) | 12752661 (20.60%) | 18911338 (30.54%) | 30253160 (48.86%) |
| LM_5 | 61765432 (100.00%) | 14807086 (23.97%) | 18588194 (30.09%) | 28370152 (45.93%) | 61773448 (100.00%) | 13861748 (22.44%) | 16884972 (27.33%) | 31026728 (50.23%) |
| Xu142_0 | 61647376 (100.00%) | 13577795 (22.02%) | 19874033 (32.24%) | 28195548 (45.74%) | 61657356 (100.00%) | 12575838 (20.40%) | 18085419 (29.33%) | 30996099 (50.27%) |
| Xu142_5 | 61687317 (100.00%) | 14171993 (22.97%) | 18580463 (30.12%) | 28934861 (46.91%) | 61693658 (100.00%) | 13344441 (21.63%) | 17114688 (27.74%) | 31234529 (50.63%) |
| Xu142fl_0 | 61781049 (100.00%) | 12945999 (20.95%) | 20304688 (32.87%) | 28530362 (46.18%) | 61790097 (100.00%) | 11941365 (19.33%) | 18705545 (30.27%) | 31143187 (50.40%) |
| Xu142fl_5 | 61391142 (100.00%) | 12133557 (19.76%) | 20932113 (34.10%) | 28325472 (46.14%) | 61407190 (100.00%) | 11322230 (18.44%) | 19582370 (31.89%) | 19582370 (31.89%) |
