# Supplementary material for: Transcriptomic repertoires depict the initiation of lint and fuzz fibres in cotton (Gossypium hirsutum L.)
Source: Plant Biotechnol J. 2017 Oct 18;16(5):1002–12. doi: 10.1111/pbi.12844 (PMC5902776; doi:10.1111/pbi.12844)
Supplement: Supplementary file 3 — Table S2 The total novel transcripts of each sample. [file PBI-16-1002-s006.docx]

Table S2 The total novel transcripts of each samples.

| Sample Name | Total Novel Transcripts | Coding Novel Transcripts | Noncoding Novel Transcripts |
| --- | --- | --- | --- |
| Xu142*fl*_0 | 46377 | 28236 | 18141 |
| LL_0 | 43878 | 27954 | 15924 |
| LM_0 | 46263 | 28106 | 18157 |
| LF_0 | 47583 | 28044 | 19539 |
| Xu142_0 | 43239 | 27673 | 15566 |
| Xu142*fl*_5 | 42881 | 27314 | 15567 |
| LL_5 | 40204 | 27368 | 12836 |
| LM_5 | 40753 | 28037 | 12716 |
| LF_5 | 37717 | 26468 | 11249 |
| Xu142_5 | 39926 | 28277 | 11649 |
